# TM-MC: a database of medicinal materials and chemical compounds in Northeast Asian traditional medicine

**DOI:** 10.1186/s12906-015-0758-5

**Published:** 2015-07-09

**Authors:** Sang-Kyun Kim, SeJin Nam, Hyunchul Jang, Anna Kim, Jeong-Ju Lee

**Affiliations:** Mibyeong Research Center, Korea Institute of Oriental Medicine, Daejeon, South Korea; Biomedical Knowledge Engineering Laboratory, Seoul National University, Seoul, South Korea; Human Derived Material Center, Korea Research Institute of Bioscience and Biotechnology, Daejeon, South Korea

**Keywords:** Medicinal material, Herb, Chemical compound, Ingredient, Traditional medicine, Northeast Asia, Chromatography, Curation, Database

## Abstract

**Background:**

In traditional medicine, there has been a great deal of research on the effects exhibited by medicinal materials. To study the effects, resources that can systematically describe the chemical compounds in medicinal materials are necessary. In recent years, numerous databases on medicinal materials and constituent compounds have been constructed. However, because these databases provide differing information and the sources of such information are unclear or difficult to verify, it is difficult to decide which database to use. Moreover, there is much overlapping information. The aim of this study was to construct a database of medicinal materials and chemical compounds in Northeast Asian traditional medicine (TM-MC), for which medicinal materials are listed in the Korean, Chinese, and Japanese pharmacopoeias and information on the compound names of medicinal materials can easily be confirmed online.

**Description:**

To provide information on the chemical compounds of medicinal materials, chromatography articles from MEDLINE and PubMed Central were searched. After chemical compounds of medicinal materials were extracted by manually investigating the full-text of articles, a database of information on about 14,000 compounds from 536 medicinal materials was built. The database also provides links to the articles from which each medicinal material and chemical compound were extracted.

**Conclusion:**

TM-MC database provides information on medicinal materials and their chemical compounds from chromatography articles in MEDLINE and PubMed Central. Researchers can easily check relevant information through the links to articles.

## Background

In the field of traditional medicine in Northeast Asia, several medicinal material databases including information on both medicinal materials and their chemical compounds have been constructed from records in the classical literature or from the results of modern research.

The traditional Chinese medicine systems pharmacology database and analysis platform (TCMSP) [1] contains information on 499 herbs with 29,384 ingredients, 3311 targets, and 837 associated diseases. The herbal ingredients were gathered during an extensive literature search, but it is not clear which works from the literature were used. The traditional Chinese medicines integrated database (TCMID) [2] provides TCM information for 46,914 formulas, 8159 herbs and 25,210 herbal ingredients and information linking them with modern medicine, including drugs and diseases. Information about the herbs and herbal ingredients was extracted from the TCM-ID [3], TCM@Taiwan [4], and a book called the Encyclopedia of traditional Chinese medicines [5]. The traditional Chinese medical literature analysis and retrieval system (TCMLARS) [6] provides search and analysis functions for biomedical journal articles published in China. It has metadata such as titles, authors, and abstracts of articles, as in MEDLINE, and includes information on the pharmacology and compatibility of TCM herbs. TCM online [7] is a database constructed by the information institute of the TCM academy, which provides integrated access to databases of patient records, TCM medicines, traditional Chinese drugs, TCM literature, and traditional Tibetan drugs. TCMLARS and TCM online provide information on chemical compounds for medicinal materials, but the sources of the information are not clear. The chemical database of traditional Chinese medicine (CHEM-TCM) [8] is comprised of two databases. One is a database providing information on 8264 chemical constituents from the 240 medicinal materials most commonly used in China. The other is a database on the target specificity of bioactive plant compounds. The former, in particular, relies on the extraction of data from several books [9–13]. The traditional Chinese medicine database (TCMD) [14] was constructed from a book [11] that includes information on Chinese medicines, original plants, and bioactive compounds. It provides details of 6800 molecular compounds isolated from more than 1540 species of natural plants used in TCM, as well as source plants, herbs, animals, and fungi. The 3D structural database [15, 16] includes information on biochemical components extracted from medicinal materials in TCM. The database contains records for 2073 TCM herbs from 298 families and 10,564 records of herbal components. It also includes optimized 3D molecular structures for at least 90 % of the components. Approximately 80 % of the records are from a survey of the literature published since 1980. The traditional oriental medicine database (TradiMed) [17] contains information on medicinal materials and formulas, including chemical compounds, disease classifications and clinical case studies, but the sources of the information are not clear.

Besides traditional medicine databases in the Northeast Asian region, there are many databases for traditional medicine in the world, such as NuBBE [18], CamMedNP [19], ConMedNP [20], AfroDb [21], p-ANAPL [22], AfroCancer [23], NPACT [24], and BioPhytMol [25]; these databases are open to the public for use. These are databases of natural products derived from plants that grow naturally in relevant regions. NuBBE is a database of medicinal plants in Brazil; CamMedNP, ConMedNP, AfroDb, p-ANAPL, and AfcroCancer contain information on medicinal plants in Africa. Further, NPACT and BioPhytMol are databases for anti-cancer natural products and anti-mycobacterial natural products, respectively. The world has a great diversity of species, and it is common that the same plant will have different scientific names and lists of constituent compounds depending on the region in which it grows. Therefore, the contents of these databases are quite different from those in Northeast Asia.

There is thus a great deal of data on medicinal materials and their chemical compounds in the medicinal material or natural product databases in the world. However, there are several obstacles to using the existing databases to obtain information on traditional medicinal materials used in Northeast Asia. First of all, most of the existing natural product databases either do not provide information on the medicinal materials of Northeast Asia [18–23], or do so without separately categorizing such information [24, 25]. In the meantime, while the medicinal material databases of the Northeast Asian region provide information on the medicinal materials of Northeast Asia and their chemical compounds, there are three difficulties to making use of the information as well.

First, because medicinal material databases provide differing information on the constituent compounds of medicinal materials, database users must compare information from each database and select the database to be used. However, it is difficult to decide which database to use because sources of the information are unclear or difficult to verify. TCMSP, TCMLARS, TCM online, and TradiMed do not state the sources from which they gathered their information. TCMID, CHEM-TCM, TCMD and the 3D structural database extracted data from published books, but it is not convenient for users of such databases to confirm their sources. Second, TCMLARS, CHEM-TCM, TCMD, the 3D structural database, and TradiMed are not available to the public. Third, there is much overlapping information about the various medicinal materials in these databases. Some databases (*e.g.*, TCMID, TCMD, and the 3D structural database) provide information on over 1000 medicinal materials, but do not distinguish synonyms for certain medicinal materials. In fact, the total number of unique medicinal materials included in the Korean, Chinese, and Japanese government-published pharmacopeias actually came to fewer than 1000.

The aim of this study was to construct a database of medicinal materials and chemical compounds in Northeast Asian traditional medicine (TM-MC), for which medicinal materials are listed in the three Northeast Asia national pharmacopoeias, and for which information on the compound names of medicinal materials can easily be confirmed in PubMed online. To achieve this goal, we searched chromatography articles from MEDLINE and PubMed Central (PMC) for medicinal plant materials. Korean medical doctors and biologists manually extracted information on the chemical compounds of medicinal materials by reading full-texts of the journal articles.

## Construction and content

Information on medicinal materials and their chemical compounds was extracted from biomedical articles contained in MEDLINE and PMC. MEDLINE is a journal citation database provided by the National Library of Medicine (NLM) and contains about 24 million abstracts for biomedical journal articles. PMC is a free digital database for about 3.1 million full-text biomedical journal articles provided by NLM. Abstracts from MEDLINE journal articles were obtained using Entrez programming utilities (http://www.ncbi.nlm.nih.gov/books/NBK25500), and the journal articles from the PMC were downloaded from (http://www.ncbi.nlm.nih.gov/pmc/tools/ftp). In this study, the curated journal articles were downloaded on December 27, 2014.

A single medicinal material can be called by various names. Despite the existence of binomial nomenclature, which is a formal system for giving names to species of living things, each country or community has its own collection of common names. In order to obtain objectively proven names of medicinal materials in our database, we used Latin names, common names, and scientific names of medicinal materials included in the Korean, Chinese, and Japanese pharmacopoeia. After excluding minerals and animals from the medicinal materials mentioned in the three pharmacopoeias, only medicinal plant materials were used for the searches in MEDLINE and PMC.

**Fig. 1:**
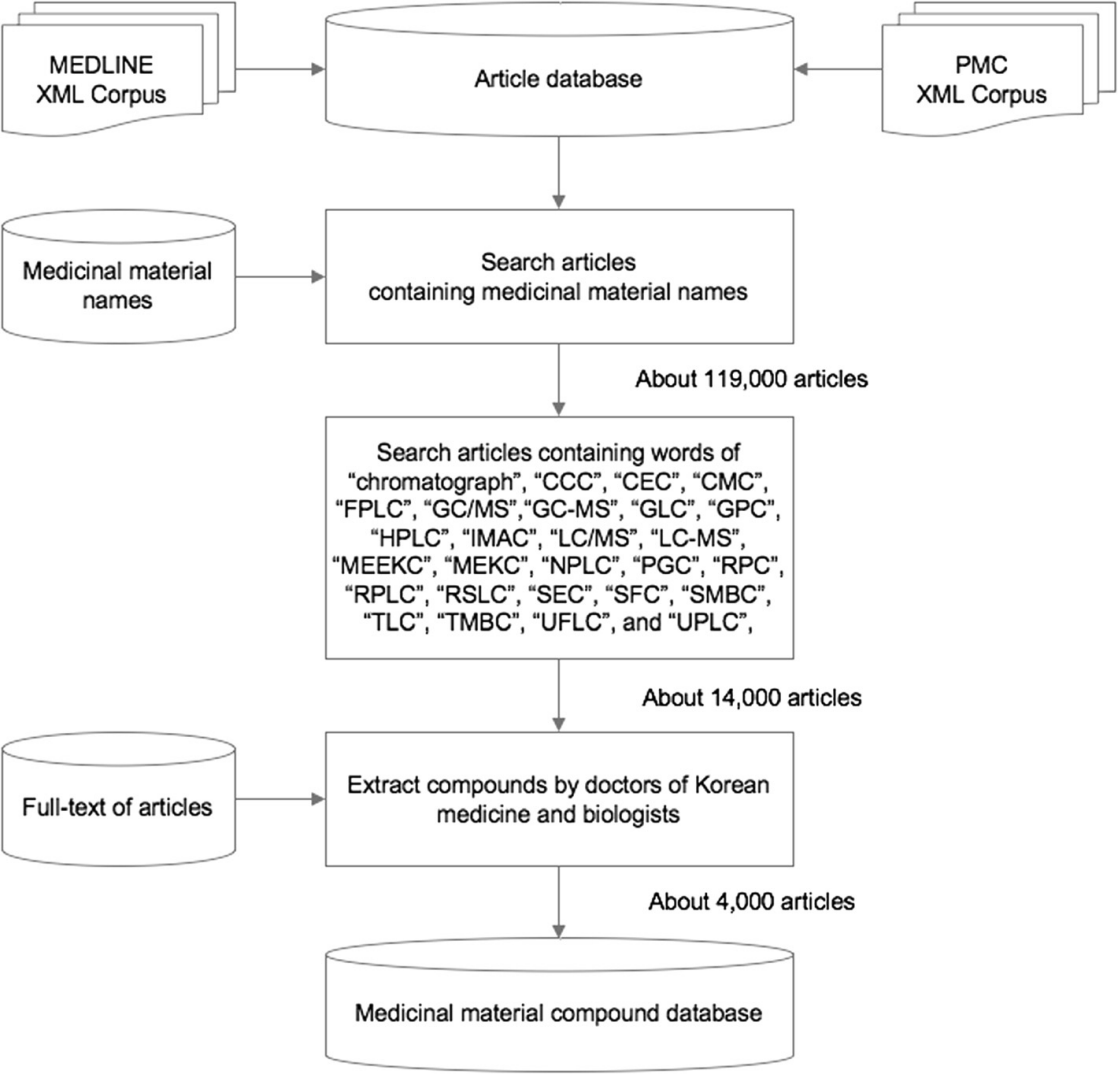
Process for constructing the TM-MC database

Figure 1 shows the overall process for constructing our TM-MC database. First, we constructed an article database with the XML corpus of MEDLINE and PMC. After texts were extracted from the titles, abstracts, and bodies of the articles in the corpus, they were indexed using Apache Lucene [26] and then stored in the article database. Articles containing medicinal materials were searched with the names of medicinal materials. Among the searched articles, chromatography articles were filtered with the following words: “chromatograph”, “CCC”, “CEC”, “CMC”, “FPLC”, “GC/MS”, “GC-MS”, “GLC”, “GPC”, “HPLC”, “IMAC”, “LC/MS”, “LC-MS”, “MEEKC”, “MEKC”, “NPLC”, “PGC”, “RPC”, “RPLC”, “RSLC”, “SEC”, “SFC”, “SMBC”, “TLC”, “TMBC”, “UFLC”, and “UPLC”. Finally, Korean medical doctors and biologists read these articles in PubMed and manually extracted information on the constituent compounds of the medicinal materials. If there was a link to full-text content from PMC or publisher web sites, the full-text was also perused. Some of these articles included chromatograms but not others. Those that did not include chromatograms were either in the form of abstracts or cited articles with chromatograms as references. It should be noted that there were some cases in which authors used different names for the same compound. If the articles contained the chemical structure or the chromatogram, readers were able to determine whether the two compounds were the same. However, distinguishing between two compounds was difficult when the articles made references by name only. In addition, it was not easy to decide which of the names to use in standardization. In this paper, because a basis for standardizing compound names was lacking, the names were retrieved as they were used in existing studies, despite the possibility of repetition.

## Utility and discussion

After information on the compound names of medicinal materials was extracted from about 4000 journal articles in MEDLINE and PMC, about 14,000 chemical compounds from 536 medicinal materials were identified. All data can now be searched or downloaded at <http://informatics.kiom.re.kr/compound>. The information on the chemical compounds of all medicinal materials in our database is linked to the articles from which the compound names have been extracted, and users can easily obtain the relevant information through these links. In general, articles can contain inaccurate information. Though these links, researchers can verify whether the information is accurate. In addition, it is also possible to verify the data quality of our database.

### Web interface

All information on medicinal materials and chemical compounds that was compiled in the present study are listed on the “Browse” menu on our website. This menu is divided into tabs so that it is possible to see all the information contained on the database. Detailed information can be seen in a new window by clicking on the “More detail” link to the right of each item.

**Fig. 2:**
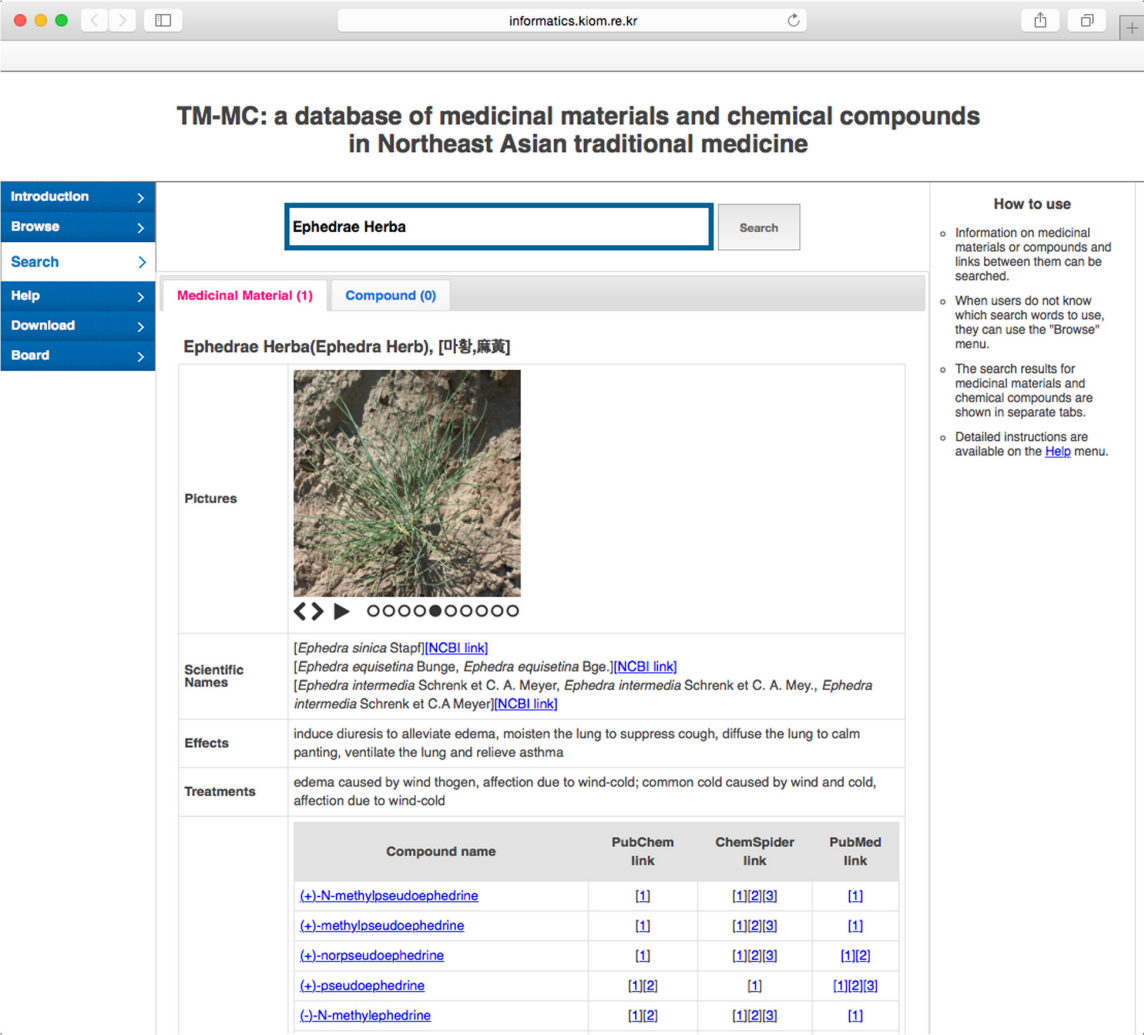
Result of search for the keyword “Ephedrae Herba”

In addition to viewing lists of all the medicinal materials and chemical compounds, users can also directly search the information using the “Search” menu. When search terms are entered in the search field at the top, a maximum of ten medicinal materials and chemical compounds that include the search terms entered are recommended, thus making searching convenient. The search results are shown at the bottom, with the medicinal materials and chemical compounds separated by tabs, together with the number of search results. Figure 2 provides an example of a search for a medicinal material called “Ephedra herba”. The name of the medicinal material is shown in the form of the “Latin Name (common name), [Korean name, Chinese name]” in the medicinal material tab, and pictures, scientific names, effects, treatments, and constituent compounds are under the name of the medicinal material. Additional details such as scientific names and information on effects and treatments comes from the medicinal material ontology built by Jang *et al.* [27]. Compared to other databases [1, 2, 6–8, 14, 15, 17], this additional information is not yet sufficient, such that users might need to search other databases at the present time. In the future, we will update our database to provide more comprehensive information on medicinal materials. A link to the NCBI Taxonomy (http://www.ncbi.nlm.nih.gov/taxonomy) is next to the scientific name, thus allowing users to obtain additional information on the plant species of the medicinal material. When a chemical compound is clicked, the name of that compound is searched again. Next to the name of the chemical compound, links to compounds in PubChem and ChemSpider, and to PubMed articles from which both the medicinal material and its chemical compound have been extracted are displayed.

**Fig. 3:**
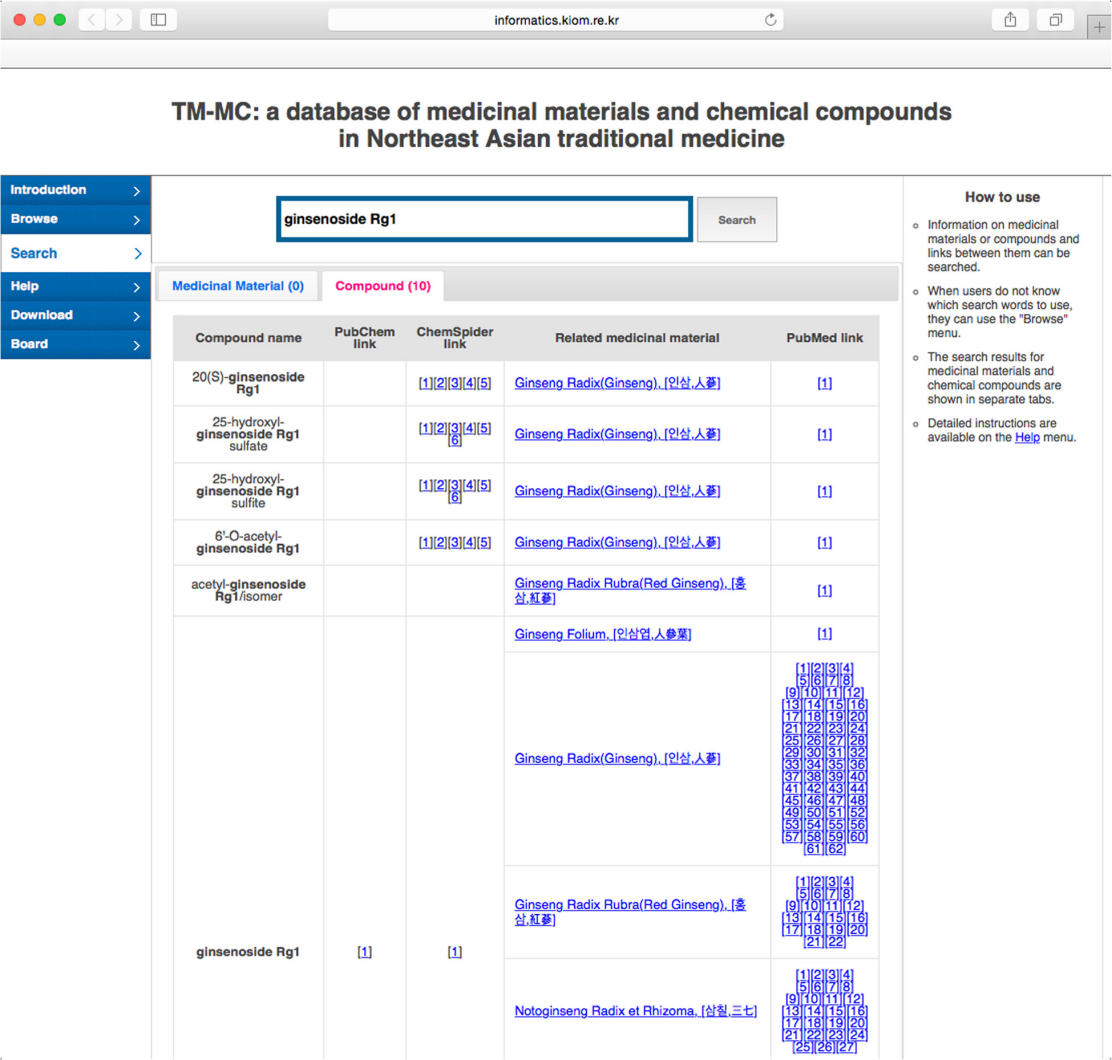
Result of search for the keyword “ginsenoside Rg1”

Figure 3 shows an example of a search for a chemical compound called “ginsenoside Rg1.” In the chemical compound tab, a list of chemical compounds including the one being searched is shown. Next to the name of each chemical compound are links to PubChem and ChemSpider, to a list of medicinal materials with the chemical compound as a constituent compound, and to PubMed papers from which information on the chemical compounds of the medicinal materials has been extracted. As in the medicinal material tab, clicking on a medicinal material prompts another search for the name of that medicinal material.

### Downloading the database contents

**Fig. 4:**
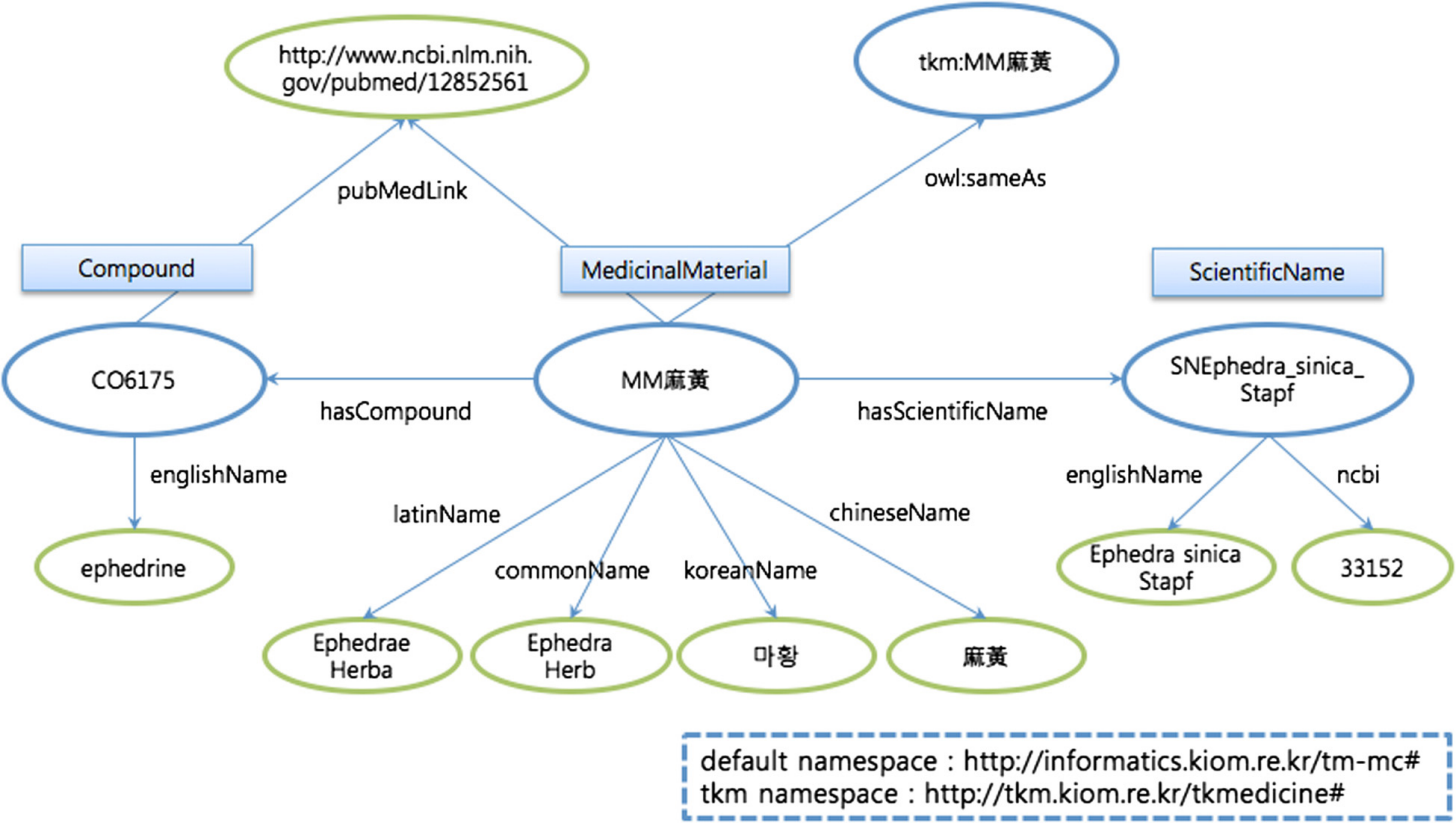
Example of a RDF graph for the Ephedra Herb in the ontology

All the information on the compound names of the medicinal materials can be downloaded as an ontology file, written in the Web Ontology Language (OWL) using the RDF/XML syntax, from the “Download” menu on our site. Many of medicinal materials in our study are identical to the medicinal materials included in the medicinal material ontology built by Jang *et al.* Therefore, the medicinal materials in our study and those in the study of Jang *et al.* are connected by the “sameAs” relationship of OWL. Figure 4 shows a part of a Resource Description Framework (RDF) graph for the Ephedra Herb in our ontology. Blue oval nodes represent objects, and green oval nodes denote data values. The rectangular shapes show the class names of the nodes.

In addition, the contents of our database can be downloaded as a single Excel file. As Excel is a program that many users feel familiar with, users will be able to access and utilize the contents of our database more easily.

### Additional chemical information

In many articles, only compound names are presented and their chemical structures are not available. To help users obtain the chemical information, including the synonyms, molecular formulas, and chemical structures of compounds, we link the compound names with the compound IDs of the PubChem Compound database [28]. The links were created using a filtered list of synonyms from the PubChem Compound database. (ftp://ftp.ncbi.nlm.nih.gov/pubchem/Compound/Extras/CID-Synonym-filtered.gz) We also provide the ChemSpider IDs as a query result of the ChemSpider Search API with the compound name. (http://www.chemspider.com/Search.asmx?op=SimpleSearch) Users can obtain additional information on chemical compounds through these links. Because a single compound name can be connected to several PubChem and ChemSpider IDs, each compound name has at least 0 or more links. In the current database, many compound names are not linked to compounds in the PubChem or ChemSpider database. They are either not registered in either PubChem or ChemSpider or are registered under different names despite being the same compound. In the future, links will be created by adding synonyms to the relevant compounds in the PubChem or ChemSpider database.

### Database updates and future plans

Our database contains information on the constituent compounds of medicinal materials extracted from articles on MEDLINE and PMC, for which general research involved the use of the chromatographic method. The chromatographic method is the general method for separating mixtures. When studies using this method were curated, it was found that our database provided information on the constituent compounds of the largest number of medicinal materials, with the exception of databases that provide overlapping information. However, to perfect our database, it will be necessary to consider a different screening method or to review all articles containing medicinal materials.

Unlike the number of medicinal materials, the number of chemical compounds currently on our database is smaller than those on other databases. This is because other databases extracted constituent compounds from books or academic papers published in Chinese. However, such data were not included in the construction of our database due to issues such as accessibility and language. Moreover, other databases do not provide information on chemical compounds extracted from recently published articles because they extracted data from books published at the latest several years ago. On the contrary, our database has the advantage of providing information on the chemical compounds of medicinal materials from the latest articles. In addition, through links to PubMed articles, our database allows users to verify their quantitative analyses of the constituent compounds of medicinal materials. Although our database currently provides only links, it will in the future provide all information on chemical compounds.

In the future, our database will periodically curate articles added to the MEDLINE and PMC database, thus updating information on the constituent compounds of medicinal materials. In addition, our database will consider other screening methods step by step, thus providing information on even more medicinal materials and constituent compounds.

## Conclusions

Our TM-MC database provides information on medicinal materials and their chemical compounds and links to articles from which information on both medicinal materials and constituent compounds were extracted. Use of this database makes it possible for researchers to determine what constituent compounds a medicinal material contains, and allows them to easily check relevant information through links to articles and other databases.

### Availability and requirements

The database is accessible at http://informatics.kiom.re.kr/compound. The database is open access and has no restrictions.

## Abbreviations

CCC: Countercurrent chromatography
CEC: Capillary electro chromatography
CMC: Cell membrane chromatography
FPLC: Fast protein liquid chromatography
GC: Gas chromatography
GLC: Gas liquid chromatography
GPC: Gel permeation chromatography
HPLC: High performance liquid chromatography
IMAC: Immobilized metal affinity chromatography
MEEKC: Microemulsion electrokinetic chromatography
MEKC: Micellar electrokinetic capillary chromatography
MS: Mass spectrometry
NLM: National Library of Medicine
NPLC: Normal phase liquid chromatography
OWL: Web Ontology Language
PGC: Pyrolysis gas chromatography
PMC: PubMed Central
RDF: Resource Description Framework
RPC: Reversed-phase chromatography
RPLC: Reversed phase liquid chromatography
RSLC: Rapid separation liquid chromatography
SEC: Size exclusion chromatography
SFC: Capillary supercritical fluid chromatography
SMBC: Simulated moving bed chromatography
TLC: Thin layer chromatography
TMBC: True moving bed chromatography
TM-MC: Database of medicinal materials and chemical compounds in Northeast Asian traditional medicine
UFLC: Ultra fast liquid chromatography
UPLC: Ultra performance liquid chromatography
